# Parathyroid carcinoma in a 30-year-old man: a diagnostic and management challenge

**DOI:** 10.1186/1477-7819-11-83

**Published:** 2013-04-08

**Authors:** Sze-How Ng, Brian Hung-Hin Lang

**Affiliations:** 1 Breast & Endocrine Unit, Department of Surgery, Kuala Lumpur Hospital, Kuala Lumpur, Malaysia; 2 Division of Endocrine Surgery, Department of Surgery, Queen Mary Hospital, 102 Pokfulam Road, Hong Kong, SAR, China

**Keywords:** Parathyroid carcinoma, Hypercalcemia, Locoregional recurrence

## Abstract

Parathyroid carcinoma is a rare endocrine malignancy, accounting for less than 1% of cases of primary hyperparathyroidism. Patient-related factors such as age and sex, as well as the biological features and management of the cancer, influence mid-term and long-term survival. We report a case of a young man with an unusual presentation of parathyroid carcinoma. The patient presented with left thigh swelling, which had been present for 6 months without other symptoms of hypercalcemia. On computed tomography scan a hypodense lesion, 30 × 20 × 20 mm in size, was seen in the posterior thyroid. There was no evidence of cervical lymphadenopathy or local infiltration. On a Sestamibi scan, a hot spot was seen in the lower pole of left thyroid lobe. Cervical neck exploration was performed. The patient subsequently underwent surgery and a parathyroid tumor was excised. The tumor was adherent to the thyroid capsule, but there was no evidence of invasion. After surgery, the patient’s calcium and parathyroid hormone levels normalized, but histology confirmed parathyroid carcinoma with capsular and vascular invasion. The patient was offered reoperation, but declined, and developed recurrent parathyroid carcinoma 2 years later. In this report, we aim to present the challenges in managing parathyroid carcinoma and discuss factors that might contribute to future locoregional recurrences. This case also highlighted several issues, including the challenge of ascertaining the diagnosis before surgery and the dilemma of reoperation after simple excision.

## Background

Parathyroid carcinoma (PC) is a rare endocrine malignancy, accounting for less than 1% of cases of primary hyperparathyroidism [1,2]. In addition, PC is a rare endocrine-related malignancy prone to locoregional recurrence (LRR), despite curative resection [1,2]. Age, sex, histopathologic features and type of resection have all been shown to influence LRR [3]. In this report, we aim to present the challenges in managing PC, and discuss some factors that might contribute to future LRR.

## Case presentation

A 30-year-old man presented with a 6-month history of non-traumatic swelling in his left thigh. Radiography of the left femur showed an osteolytic mid-shaft lesion without any fracture. There were no other symptoms such as weakness, fatigue, nausea, vomiting, constipation, dyspepsia, headache, polyuria, or abdominal pain.

Laboratory investigations showed that the patient had raised levels of adjusted calcium (3.55 mmol/l; normal range 2.24 to 2.63 mmol/l), phosphate (0.44 mmol/l; normal range 0.88 to 1.43 mmol/l) and parathyroid hormone (PTH) (2,187 pg/ml; normal range 10 to 66 pg/ml).

**Figure 1:**
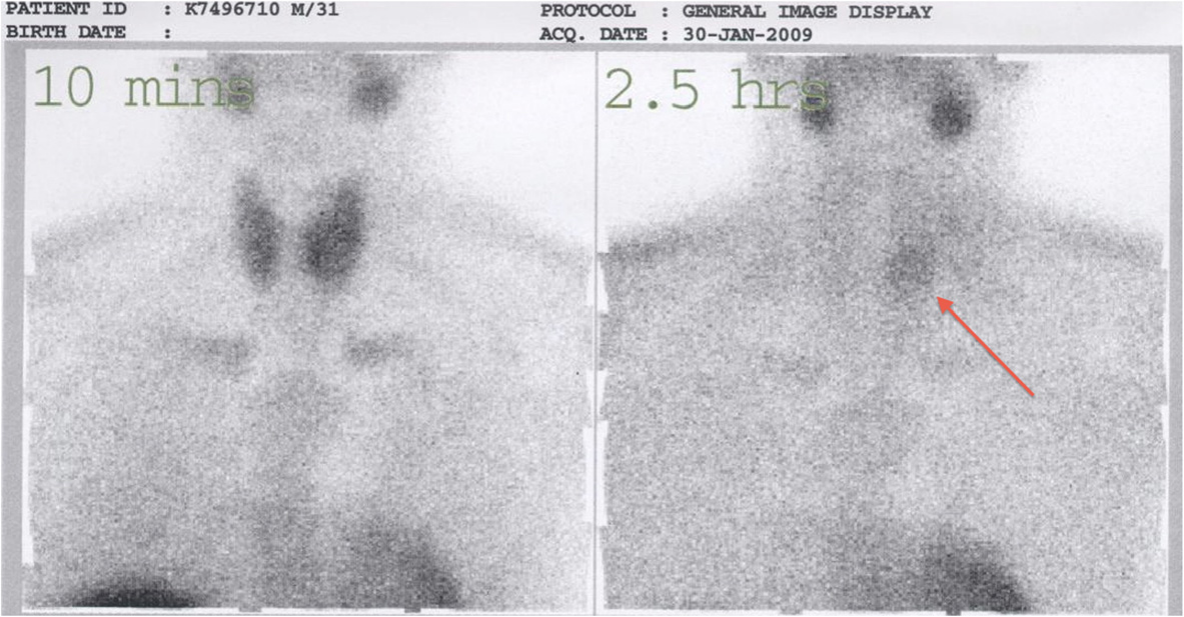
**A delayed image (2.5 hours) of the Sestamibi scans.** There was focal retention of tracer over the left middle or lower pole of the thyroid (arrow).

**Figure 2:**
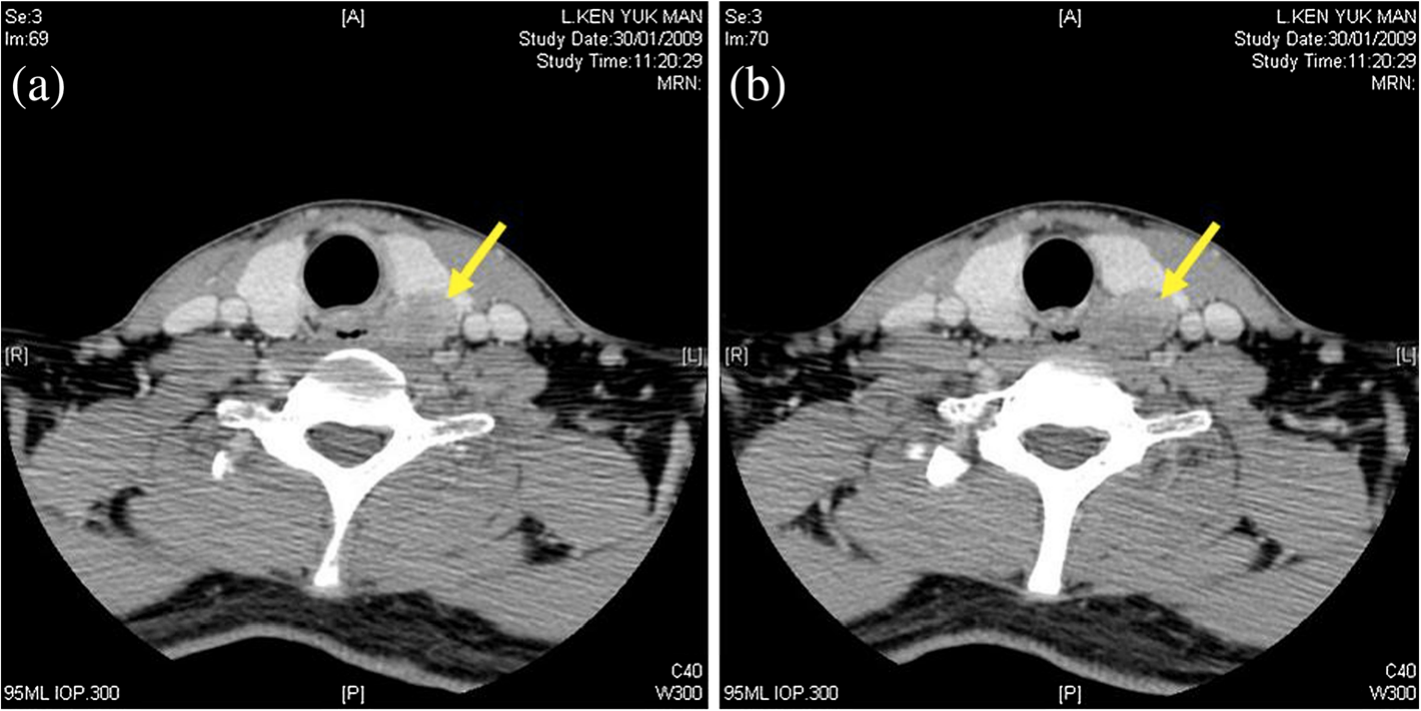
**(a,b). Axial computed tomography scan of the neck.** A hypodense lesion 30 × 20 × 20 mm in size was visible in the posterior thyroid (arrow) without cervical lymphadenopathy or local infiltration.

**Figure 3:**
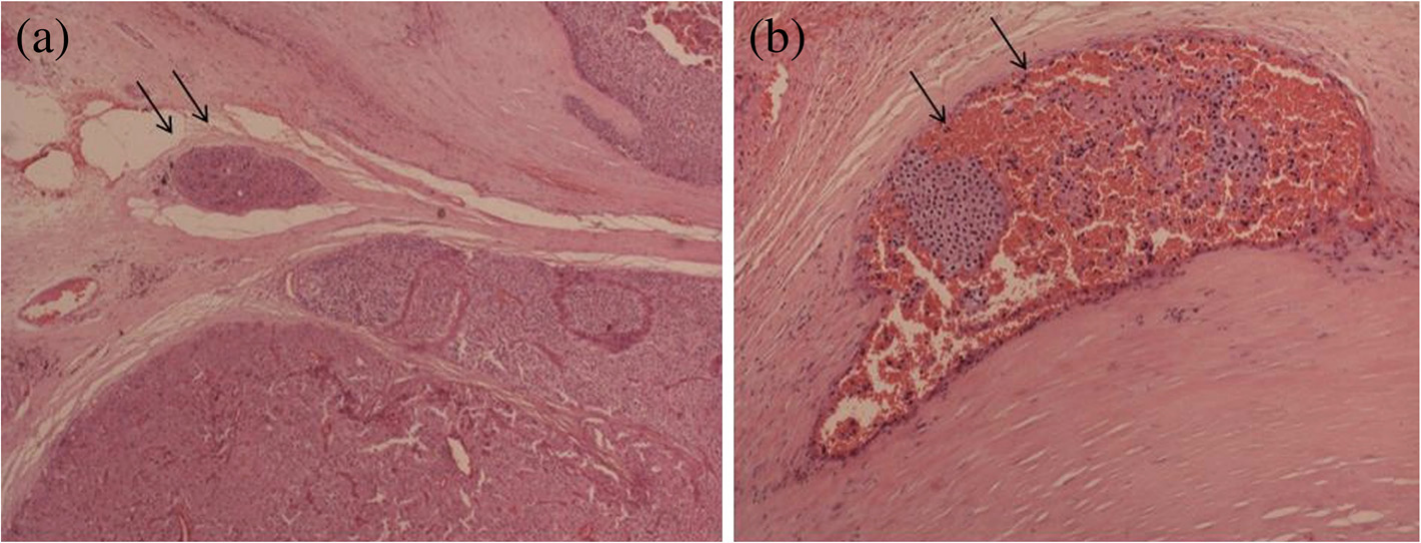
**(a,b). Vascular invasion by the tumor cells.** Hematoxylin and eosin, original magnification (**a**) ×10; (**b**) ×40.

On clinical examination, a small mass was palpable over the left thyroid gland. Sestamibi scan showed a hot spot localized in the left mid-pole of the thyroid (Figure 1). On a computed tomography (CT) scan of the neck, a hypodense lesion, 30 × 20 × 20 mm in size was seen in the posterior the thyroid, without cervical lymphenopathy or local infiltration (Figure 2). Cervical neck exploration was performed subsequently. The patient underwent surgery, during which a large parathyroid tumor measuring 45 × 30 × 25 mm and weighing about 15 g was excised. The tumor was adherent to the thyroid capsule, but there was no evidence of invasion. An intra-operative parathyroid hormone (IOPTH) assay showed a drop of 90% in PTH level 10 minutes after the excision of the pathological gland. After the operation, the patient’s calcium and PTH levels normalized, but histology confirmed PC with capsular and vascular invasion (Figure 3). In view of this, the patient was offered reoperation, but he refused and did not attend for follow-up.

Two years later, the patient re-presented. He was found to have palpable neck swelling, and laboratory investigations showed hypercalcemia and raised PTH. An ipsilateral lobectomy and central lymph-node dissection were performed. IOPTH once again showed a drop in PTH (this time of more than 50%) 10 minutes after the procedure. The histology results confirmed recurrent PC, but all six lymph nodes were negative for metastasis. The patient’s serum calcium and PTH level normalized after the second operation. He received a course of external radiotherapy, and has remained disease-free during a follow-up period of 18 months.

## Discussion

Unlike parathyroid adenoma, PC affects both sexes equally, and patients are around 10 years younger than those with parathyroid adenoma [4]. The current patient was a man in his early 30s, which is not the typical presentation of PC. In a recent study of 330 patients, male sex, young age, and higher calcium levels were adverse clinical prognostic factors, thus based on this, our patient would be considered at high risk [3,5].

This case highlights several issues, including the challenge of ascertaining the diagnosis of PC before surgery, and the dilemma of reoperation after simple excision. It is difficult to differentiate PC from adenoma, and pathological findings are significantly associated with outcomes [2]. The criteria proposed by Schantz and Castleman seem to be the key features distinguishing carcinoma from benign counterparts. These include a trabecular pattern, mitotic figures, thick fibrous bands, and capsular and blood-vessel invasion [2,6]. Fine-needle aspiration cytology would not be able to distinguish adenoma from carcinoma, as the diagnosis requires histological morphology and criteria [7]. Although extremely high PTH and calcium level (>3.5 mmol/L) may be the first indication of malignancy, a wide overlap between benign and malignant tumors has been reported [8]. In our case, the PTH level was extremely high, but the patient’s symptoms and calcium level were not suggestive of malignancy. Other features such as osteitis fibrosa cystica, a palpable neck mass, a high depth:width ratio on ultrasonography and CT tomography are important features of PC [2].

Based on the scintigraphy and IOPTH assay, removal of the parathyroid tumor in this case was successful. Although more than 90% of PCs are functional and scintigraphy may be a valid tool for tumor localization, it tends to fail in localizing LRR and metastases [9]. In addition, the role of IOPTH in managing PC is still doubtful, even though it has been proven as an effective and safe method for the treatment of primary hyperparathyroidism [10]. Although *en bloc* resection is often recommended for PC, it is possible only in 10 to 15% of cases because ascertaining the diagnosis of PC is difficult and requires a high index of suspicion [2]. In this case, we performed an *en bloc* resection with ipsilateral lobectomy for the second operation, as the choice of surgical procedures significantly influences the outcome. Carrying out local excision only compared with any form of wider excision carries a risk 1.5-fold to 2-fold higher for recurrence and death both at 5 years and overall [3].

## Conclusion

PC is a very rare disease and making the diagnosis of PC is often difficult. This leads to delayed diagnosis when the tumor is already palpable. In addition, prognosis is not predictable because after the malignancy is confirmed, recurrences occur in most cases. In the setting of a suspected PC, neck exploration must be performed with an *en bloc* resection of the tumor with ipsilateral thyroid lobectomy. A high index of suspicion and a moderate enhancement of the surgical approach including systemic central node dissection may improve the outcome for PC.

## Consent

Written informed consent was obtained from the patient for publication of the case report and any accompanying images. A copy of written consent is available for review by the Editor-in-Chief of this journal.

## Abbreviations

CT: Computed tomography; IOPTH: Intra-operative parathyroid hormone assay; LRR: Locoregional recurrence; PC: Parathyroid carcinoma; PTH: Parathyroid hormone.

## Competing interests

The authors declare that they have no competing interests.

## Authors’ contributions

SHN was involved in the review of literature, acquisition of data and drafting and completing the manuscript. BHHL conceived the study, participated in the co-ordination and the acquisition of data, and helped to draft the manuscript. Both authors read and approved the final manuscript.
